# Hippocampal-Dependent Spatial Memory in the Water Maze is Preserved in an Experimental Model of Temporal Lobe Epilepsy in Rats

**DOI:** 10.1371/journal.pone.0022372

**Published:** 2011-07-26

**Authors:** Marion Inostroza, Elena Cid, Jorge Brotons-Mas, Beatriz Gal, Paloma Aivar, Yoryani G. Uzcategui, Carmen Sandi, Liset Menendez de la Prida

**Affiliations:** 1 Instituto Cajal, Consejo Superior de Investigaciones Cientificas, Madrid, Spain; 2 Universidad Europea de Madrid, Madrid, Spain; 3 Laboratory of Behavioral Genetics, Brain Mind Institute, École polytechnique fédérale de Lausanne, Lausanne, Switzerland; 4 Departamento de Psicología, Universidad de Chile, Santiago, Chile; McGill University, Canada

## Abstract

Cognitive impairment is a major concern in temporal lobe epilepsy (TLE). While different experimental models have been used to characterize TLE-related cognitive deficits, little is known on whether a particular deficit is more associated with the underlying brain injuries than with the epileptic condition *per se.* Here, we look at the relationship between the pattern of brain damage and spatial memory deficits in two chronic models of TLE (lithium-pilocarpine, LIP and kainic acid, KA) from two different rat strains (Wistar and Sprague-Dawley) using the Morris water maze and the elevated plus maze in combination with MRI imaging and *post-morten* neuronal immunostaining. We found fundamental differences between LIP- and KA-treated epileptic rats regarding spatial memory deficits and anxiety. LIP-treated animals from both strains showed significant impairment in the acquisition and retention of spatial memory, and were unable to learn a cued version of the task. In contrast, KA-treated rats were differently affected. Sprague-Dawley KA-treated rats learned less efficiently than Wistar KA-treated animals, which performed similar to control rats in the acquisition and in a probe trial testing for spatial memory. Different anxiety levels and the extension of brain lesions affecting the hippocampus and the amydgala concur with spatial memory deficits observed in epileptic rats. Hence, our results suggest that hippocampal-dependent spatial memory is not necessarily affected in TLE and that comorbidity between spatial deficits and anxiety is more related with the underlying brain lesions than with the epileptic condition *per se*.

## Introduction

Cognitive deficits represent a serious neuropsychological problem in people suffering from temporal lobe epilepsy (TLE) [1], [2]. While their origin still remains unknown, many factors have been proposed to contribute, including medication [3], seizures [4], [5], abnormal forms of interictal activity [6]–[9] and circuit reorganization [10]. The nature of the cognitive deficits more common in TLE is also a matter of debate. Impairment of a variety of memory types has been extensively described, with verbal, episodic and spatial working memory being mentioned [11]–[13]. In addition, other coexisting disorders, like depression, stress and anxiety, are known to exacerbate cognitive deficits in TLE [14]–[17]. Questions are open as to whether they merely concur due to several psychosocial aspects or rather there is a neurobiological basis for this comorbidity.

Chronic epilepsy induced in rats by systemic administration of lithium-pilocarpine or kainic acid reproduces most clinical and neuropathological features of human TLE [18], [19]. In adult rats, the injection of these drugs leads to a *status epilepticus* followed by a chronic period when most animals exhibit spontaneous convulsive seizures, usually weeks after injection [20]–[22]. Neuronal loss is not only present in the sclerotic hippocampus, but also affects the parahippocampal cortical regions, the thalamus, the endopiriform cortex and the amydaloid nuclei [18], [23]–[25]. Most of these structures are directly involved in different memory processes and in their modulation. However, the extent and specificity of brain lesions might depend on the model in use [25]. Hence, validation of animal models is essential for a better understanding of the basic mechanisms involved in TLE-associated cognitive deficits.

Previous studies have reported severe impairment of hippocampus-dependent spatial memory in pilocarpine-treated rats as assessed in the Morris water maze [26], [27], and in spatial memory tasks that presumably depend on the hippocampal integrity [28], [29]. In contrast, kainate-treated rats appear less impaired in similar tasks [30], [31]. Similarly, there are conflicting results regarding the performance of epileptic rats in the elevated plus maze, an unconditioned spontaneous test to measure anxiety [29], [32], [33]. Given that lesions induced by pilocarpine and kainic acid represent multifocal brain damage of different intensity and extension, these models can be used to test whether the particular expression of a cognitive deficit is related to the epileptic condition or to the underlying brain injuries.

In the present work, we investigated the relationship between the extension of brain injuries and spatial memory deficits using two different models of TLE (lithium-pilocarpine and kainic acid) from two different rat strains (Wistar and Sprague-Dawley). We specifically checked for spatial memory deficits and coexisting anxiety disorders using the Morris water maze and the elevated plus maze, and further assessed the type and extension of brain lesions and cell loss by combining magnetic resonance imaging (MRI) and neuronal immunostaining. We found major differences between LIP- and KA-treated rats regarding the extension of brain lesions, spatial memory deficits and anxiety. We discuss our findings in the context of the type of memory disruption in TLE and the relationship with anxiety. Our data demonstrate that the lesional patterns have to be carefully considered when evaluating cognitive deficits in experimental models of TLE.

## Materials and Methods

All procedures met the European guidelines for animal experiments (86/609/EEC). Protocols were approved by the Ethics Committee at the Instituto Cajal for the application grants BFU2009-07989 and MemStick (201600).

### Subjects

Adult male Wistar and Sprague-Dawley rats weighing 180–200 g were obtained both from the Harlan Laboratories and from our animal facilities (Instituto Cajal). Rats were housed in groups of four animals per cage under controlled conditions (temperature of 22±2°C and 12∶12 light–dark cycle, lights on at 7 a.m). The animals were given free access to food and water.

### Lithium-pilocarpine (LIP) treatment

Rats from both strains were i.p. injected with pilocarpine hydrochloride 12–24 hr after the injection of lithium chloride (127 mg/kg, i.p. [19]). Between one and four doses of 10 mg/kg pilocarpine were injected every 30 min until the *status epilepticus* was reached. The *status* was defined as a condition of continuous seizures lasting longer than 30 min. Diazepam (4 mg/kg, i.p.) was injected 1 hour after the *status* and repeated during the following 24 hours if convulsive behaviors persisted. Animals were i.p. injected with 2.5 ml 5% dextrose several times a day, and diet was supplemented with fruit and powder milk during the following 2–3 days. Animals that did not exhibit *status* after four doses of 10 mg/kg pilocarpine were considered resistant and were excluded from the study. Mortality rate was 25% for Sprague-Dawley rats and 27% for Wistar animals.

### Kainic acid (KA) treatment

Independent sets of rats from both strains were i.p. injected with kainic acid (5 mg/kg) at hourly intervals until they reached the *status epilepticus* [34]. Between two and four doses of 5 mg/kg kainic acid were required to reach the *status* in different animals. Similar to LIP-treated rats, the convulsive *status* was interrupted by diazepam (4 mg/kg, i.p.) one hour after the start. Animals that did not exhibit *status* after four doses of 5 mg/kg kainate were considered resistant and were excluded from the study. Mortality rate was 6% for Sprague-Dawley animals and 8% for Wistar rats.

### Control groups

We defined two control groups. Control group I was composed of untreated animals that receive similar diet and housing conditions than experimental animals. Control group II was composed of rats treated with vehicle (saline) instead of pilocarpine or kainic acid and received similar treatment than the experimental group, i.e. lithium and diazepam for the lithium-pilocarpine model and diazepam for the kainate model. Control groups I and II were separated by strains. Since no statistical differences in task performance between each strain-specific control group were found, the data from control groups I and II for each strain were pooled.

### Experimental design

Epileptic rats were assigned to one of four groups depending on the treatment and strain: KA-Wistar (n = 12), KA-Sprague-Dawley (n = 16), LIP-Wistar (n = 11) and LIP- Sprague-Dawley (n = 12). Control groups consisted of n = 23 Wistar rats and n = 9 Sprague-Dawley rats. Behavioral tests typically started after about 8 weeks post-*status*, when the animals already exhibited spontaneous seizures, as scored according to the Racine scale (see below). From that moment the rats were handled for 4 days before being tested in the elevated plus maze (EPM), a test that is used to determine differences in anxiety levels. Six days after EPM testing, rats were tested in a standard version of the Morris water maze (MWM) to investigate whether the epileptic groups were able to learn a hippocampal-dependent spatial task. Behavioral tests were suspended for at least two hours for those rats exhibiting spontaneous seizures. We also considered possible pre-seizure effects by excluding from the analysis those animals experiencing at least one seizure within the next hour after completing the test. All behavioral tasks were performed between 9:00 and 14:00 hours. After completing experiments, a group of animals was submitted to MRI studies (control-Wistar n = 6, control-Sprague Dawley n = 5, KA-Wistar n = 7, KA Sprague Dawley n = 10, LIP-Wistar n = 6 and LIP-Sprague Dawley n = 8) to compare the structural differences between models. Finally, histology and neuronal immunostaining were performed to confirm the presence of hippocampal sclerosis, a pathological hallmark of TLE characterized by a particular pattern of cell loss and the sprouting of recurrent excitatory collaterals by granule cell axons known, as mossy fiber sprouting.

### Spontaneous seizures: clinical and electrophysiological characterization

Typically, rats were observed for behavioral and clinical signs of spontaneous seizures at random times between 8 a.m. and 7 p.m. during at least 20 min and 3–4 times per week starting two weeks post-injection. When no spontaneous seizure was observed, animals were continuously video-taped during 48–72 hours for seizure detection. This monitoring protocol aimed to confirm the epileptic nature of the animals before starting behavioral tests. In a group of animals we performed longer video recordings during light time periods accumulating hundreds of hours for quantification purposes (KA-Wistar n = 9, KA-Sprague-Dawley n = 6, LIP-Wistar n = 6 and LIP- Sprague-Dawley n = 9). Spontaneous seizures from these animals were scored according to Racine [35]: stage 1) orofacial automatisms, stage 2) stage 1 and head nodding, stage 3) forelimb clonus, stage 4) forelimb clonus with rearing, and stage 5) stage 3 and 4 with animal falling. Forelimb convulsions were used for obtaining a measure of convulsive seizure duration, which was estimated using videos at slow speed from the start of forelimb automatisms to the moment of posture recovery. Mean seizure grade and duration were calculated from each animal by averaging several scores from independent spontaneous seizures. Typically, between 1 and 4 spontaneous seizures were observed per rat. A grand average of the seizure grade and duration, i.e. the average of sample averages, was computed for each experimental group.

Two rats from each group were checked for electrographic seizures affecting the dorsal hippocampus using depth chronic recordings. For electrode implantation, animals were anesthetized with 1.5–2% isofluorane mixed with oxygen (400–800 ml/min) and fixed to a stereotaxic apparatus. Local field potential (LFP) recordings were obtained either from 50 µm nichrome/formvar wires (impedance 0.3–0.5 Momhs) or 16-channel silicon probes (NeuroNexus Tech; site impedance between 0.3–1.2 Momhs) implanted at the dorsal hippocampus using bone cement. Implantation coordinates were between 3.9–4.8 mm posterior to bregma and 3 mm from the midline. We adjusted the implantation depth guided by intra-operative electrophysiological recordings. The probe was isolated using vaseline to facilitate stability and to avoid contact between cement and the brain. Two screws served as a reference and ground at the occipital region. After recovering from surgery, spontaneous seizures were recorded from individual rats and classified according to the clinical signs using the Racine scale. Electrographic signals were pre-amplified using field-effect transistors (FETs) and further amplified and digitized at different sampling rates. For offline analyses, recordings were band-pass FIR filtered between 1 and 200 Hz and down-sampled at 500 Hz. After completing recordings brains were fixed in 4% paraformaldehyde for electrode placement verification. Wire electrodes were found in the dorsal CA1 region typically below the pyramidal layer at sites within the stratum radiatum. In the case of the 16-channel silicon probes, LFP signals were selected from electrodes at the stratum radiatum to match similar recording sites using wire recordings. Electrographic seizures were defined as a typical ictal pattern with discharge amplitude larger than 2.5SD baseline followed by a clear pattern of rhythmic spike-and-wave discharges at >5 Hz.

### Elevated plus maze

Anxiety-related behavior was evaluated using the EPM test. The maze consists of two opposing open arms (45×10 cm) and two enclosed arms (45×10×40 cm) that extend from a central platform (10×10 cm), elevated 50 cm above the floor. Rats were placed individually on the central region facing an enclosed arm and were allowed to freely explore the maze for 5 min. The behavior of each rat was monitored using a video camera, and movements were analyzed with a computer tracking system (Ethovision 1.90, Noldus IT). Two LIP-treated rats from each strain jumped from the apparatus and they were not included for analysis. Entries into an arm were defined when all four paws were into the corresponding arm. Measures of anxiety-related behavior included the percentage time spent in the open and closed arms, with larger time in the open arms indicating lower levels of anxiety. We also checked for the level of locomotor activity by measuring the total travelled distance.

### Morris water maze: cued learning, acquisition and retention

The Morris water maze (MWM) consists on a black circular pool (180 cm diameter, 45 cm high) filled with water (30 cm depth) at 22±1°C and virtually divided into four equivalent quadrants: north-east (NE), north-west (NW), south-east (SE) and south-west (SW). A 2-cm submerged escape platform (12 cm diameter) was placed in the middle of one of the quadrants equidistant from the sidewall and the center of the pool. The pool was surrounded by several distal visual cues. Animal behavior (latency to escape, total distance and thigmotactic swimming) was monitored by a video camera and quantified using Ethovision v1.90 (Noldus). Thigmotactic swimming, i.e., the behavior that an animal displays when swimming close to the walls of the water maze, was quantified by dividing the pool into two circles. Swimming occurring in the outer ring of the pool (20 cm wide) was defined as thigmotactic and the time spent in that ring was measured for quantification purpose.

Before starting the MWM acquisition phase, we performed a cued learning training to instruct the animals on the procedures required to learn the MWM task. For cued learning, the pool is the same as in the MWM version except that the platform is flagged above the water by approximately 12 cm and curtains are closed around the maze to avoid access to distal visual cues. To ensure that rats are using the flagged cue to locate the platform, the location of the goal and the starting point are both moved to new positions during each trial. Four trials per day were performed until the rat reached the platform in two consecutive trials with a latency of less than 20 seconds.

After completing cued learning, rats were trained with the standard MWM learning protocol consisting of 4 consecutive trials during 3 days starting randomly from one of the four quadrants each time. A trial began by placing the rat into the water facing the wall of the pool. The rat was guided to the platform if failed to escape within 90 s and was allowed to stay in the platform for 20 s maximum before returning home cage for a new trial (inter-trial interval 20 sec). On day 4, we conducted a probe trial in which the escape platform was removed from the pool and the rat was allowed to swim for 60 sec. The trial began with the rat in the quadrant opposite to the trained platform location. The time spent in each quadrant was recorded. In order to discriminate against the chance level, a ratio was defined as the percent time spent in the target quadrant over the total time spent in the target and the opposite quadrant (chance level at 50%). The acquisition learning phase lasting 3 days provides a measure of spatial learning and reference memory, while the probe trial at day 4th measures spatial memory and retrieval capabilities.

### Volumetric analysis of MRI data

Magnetic resonance imaging (MRI) was obtained from the Imaging platform at the Instituto de Investigaciones Biológicas, from the Spanish National Research Council (CSIC). Imaging was acquired using a Bruker Pharmascan system (Bruker Medical Gmbh, Ettlingen, Germany) with a 7.0-T horizontal-bore superconducting magnet, equipped with a ^1^H selective birdcage resonator of 38 mm and a Bruker gradient insert with 90 mm of diameter (maximum intensity 30 G/cm). Data were acquired using a Hewlett-Packard console (Bruker Medical Gmbh) operating on a Linux platform. Anaesthesia was induced by 4% isofluorane mixed with oxygen (1 l/min) and maintained at 2% isofluorane. Animal temperature was maintained at 37°C with a heated blanket. The physiological state of the rats was supervised using a physiological monitoring system (Biotrig, Bruker) that controlled the respiratory rate and body temperature. T2-weighted spin-echo anatomical images were acquired with a relaxation enhancement (RARE) sequence in axial orientations and the following parameters: TR  = 3000 ms, TE  = 59 ms, RARE factor  = 8, Av  = 3, FOV  = 3.8 cm, acquisition matrix  = 256×256 corresponding to an in-plane resolution of 148×148 µm^2^, slice thickness  = 1.50 mm and number of slices  = 20 for axial orientation, 12 for saggital orientation and 8 for coronal orientation.

Partial volumes from several brain structures were obtained from the MRI images by using the public domain Java-based imaging-processing program ImageJ (http://rsb.info.nih.gov/ij/). The regions of interest (ROI) were manually outlined using the corresponding section from the stereotaxic atlas [36]. For all ROIs, measures from both hemispheres and consecutive sections were combined together. The following ROIs were measured in the coronal planes running from -2 mm to about -4 mm from bregma: the dorsal hippocampus, the basolateral amygdala and the perirhinal and piriform cortex. Horizontal planes corresponding to -6 mm to -8 mm ventral to the interaural horizontal level were used to measure part of the ventral hippocampus and the entorhinal cortex. Volumetric measurements from each ROI were normalized to the brain volume, yielding a ratio that is independent of the animal's brain size.

### Histology: neuronal immunohistochemistry, Timm staining and electrode verification

After completing experiments, rats were perfused intracardially with 30 ml of phosphate buffered saline 0.1 M, pH = 7.3 (PBS) with 0.2% heparin, followed by 200 ml of 4% paraformaldehyde in PBS. The brains were then removed and postfixed by immersion for 1 hour. Coronal sections of 100 or 50 µm were cut on a vibratome. For immunostaining, free-floating sections were washed in PBS followed by incubation in 1% H_2_O_2_ for 15 min. After washing in PBS several times, sections were maintained during 1 hour in PBS containing 10% fetal bovine serum (FBS) and 0.25% Triton and then incubated overnight at 4°C in monoclonal anti-NeuN antibody (1∶1000, Bachem) diluted in PBS containing 1% FBS and 0.25% Triton. On the second day, sections were washed and incubated for 2 hours in biotinylated secondary antibody (anti-mouse IgG, 1∶200, Jackson) and for 1 hr in avidin-biotin- peroxidase complex (1∶1000, Vector) diluted in PBS-1% FBS. Afterwards, they were washed in PBS, revealed with 0.05% 3,3-diaminobenzidine and 0.01% H_2_O_2_, and mounted on slides coverslipped with glycerol and Eukitt (Fluka). We also validated the presence of mossy fiber sprouting (MFS) using the Timm staining. For Timm staining, rats were perfused with Na_2_S 0.1% in PBS 0.1 M with heparin 0.2% at 4°C before paraformaldehyde fixation. Free-floating sections (70 µm) were developed in dark, using arabig gumm, citric acid, hydroquinone and silver nitrate during at least 20 minutes. Electrode verification from animals chronically implanted were performed using coronal sections of 100 µm and the thionin staining.

### Stereological cell counting

We obtained estimates of the total numbers of NeuN-labelled cells in the CA1 and CA3 regions of the dorsal hippocampus and the lateral and basolateral amygdala of one hemisphere per rat. We used unbiased stereological methods [37] and a computer-assisted system (Stereo Investigator, MicroBrightField Bioscience) coupled to an Olympus microscope (BX51, Olympus). Neurons were counted at 100× amplification. For the dorsal hippocampus, one out of every 8 sections of 50 µm corresponding to about 2.76 mm and 4.36 mm posterior to bregma, were counted. In each hippocampal section, the CA3 and CA1 areas were delineated using a 4× objective as previously described [37]. Estimations on the neuron numbers can differ according to how boundaries between CA regions are delineated. We adopted the following criteria: a) neurons included in the CA3 region were counted from the tip of the pyramidal layer enclosed by the dentate gyrus to the CA1 border; b) the border between CA3 and CA1 was established at the point where pyramidal neurons became more compact and of smaller size; c) CA1 was delineated excluding the subiculum at the point where the narrow and tightly-packed row of neurons in the pyramidal layer gives way a wider cell layer. The size of the counting frame was 50×50 µm for grids of 120×120 µm (CA3) and 150×150 µm (CA1) and a dissector height of 10 µm, giving a coefficient of error (CE, Gundersen equation) <10% and ratio between CE^2^/CV^2^ <0.2. Neurons were counted if their somata were found within the counting frame or overlapping its top-right border. For the basolateral amygdala, one out of every 8 sections of 50 µm corresponding to about 2.76 mm and 4.08 mm posterior to bregma, were counted. The limits of the lateral and basolateral amygdala were delineated using a 4× objective and the atlas of Paxinos and Watson [36]. After careful evaluation we decided to count cells only in the lateral and basolateral amygdala because the posterocortical amygdaloid nuclei were already lesioned in most epileptic rats. Also, due to difficulties in establishing clear boundaries in many LIP-treated animals exhibiting important amygdalar shrinkage, we only performed cell counting in the KA group. The size of the counting frame was 50×50 µm for a grid of 150×150 µm and a dissector height of 10 µm, giving a CE <5% and CE^2^/CV^2^<0.04. We obtained an unbiased estimate of the total number of neurons, the volume and the neuronal density for the individual regions.

### Timm scoring

The extent of mossy fiber sprouting was evaluated blindly by three observers, using Timm-stained sections corresponding to about 5.4 mm posterior from bregma and standardized scoring procedures [38]. Briefly, mossy fiber sprouting was evaluated by rating the distribution of supragranular Timm granules (TC) at a standard location in the dorsal and the ventral hippocampus. Timm scoring scale ranges from 0 to 5 according to the following criteria: 0, no TC in the supragranular region; 1, sparse TC in the supragranular regions in a patchy distribution; 2, several TC in a continuous distribution; 3, prominent TC on a continuous distribution with occasional patches of confluent TC; 4, prominent TC that form a confluent dense laminar band and 5, a confluent dense laminar band of TC that further extends into the inner molecular layer. A mean Timm score for each rat was calculated by averaging the independently derived scores.

### Statistics

Results are reported as mean ± SEM, except otherwise indicated. Data analysis was performed using the statistical software SPSS 18.0 for Windows. Normality and homogeneity of variance was confirmed using the Kolmogorov-Smirnov test. For the water maze experiments, we used a repeated-measures ANOVA with groups (control-Wistar, control-Sprague Dawley, KA-Wistar, KA-Sprague Dawley, LIP-Wistar and LIP-Sprague Dawley) as the grouping factor and trials (trials 2–8 in the cued learning task; trials 1–12 in the acquisition phase for analyzing training and thigmotactic behavior) as repeated measures. Mean comparisons for data from the target quadrant (probe trial-MWM), the time in open arms (EPM) and the volumetric analysis of MRI (amygdala, dorsal hippocampus and ventral hippocampus) were carried out with one-way ANOVA for the six experimental groups. Tukey's HSD post hoc paired comparison tests were run on all significant effects and results were considered significant at p<0.05. Differences in seizure grade between the epileptic groups were analyzed using the Kruskal-Wallis non-parametric test. Cell counting estimates and Timm scoring were compared using non-parametric statistical analysis followed by a Mann-Whitney test. In addition, we performed a Pearson correlational analysis in a subset of animals to evaluate possible associations between the behavioral parameters obtained in the water maze and the EPM and also possible associations with the structural differences obtained by MRI.

## Results

### Seizure severity is similar in LIP- and KA-treated epileptic rats

**Figure 1:**
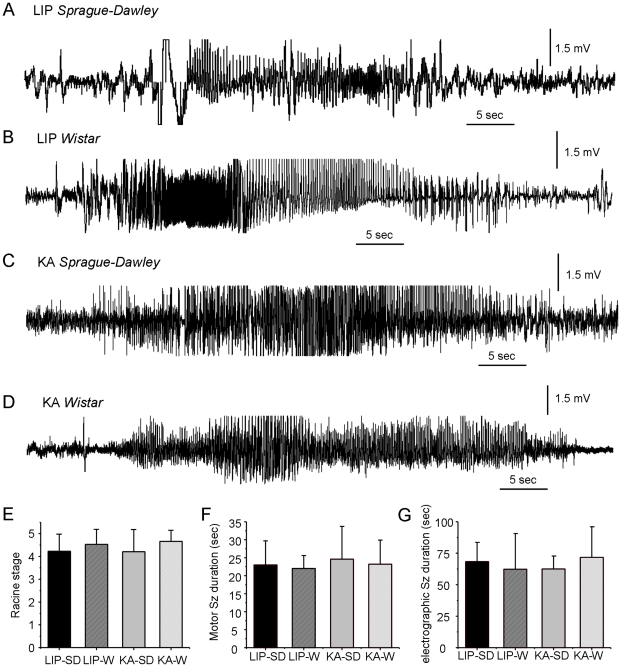
Typical electrographic seizures recorded from the dorsal hippocampus from rats treated either with lithium-pilocarpine (LIP) of kainic acid (KA). (A) LIP- Sprague-Dawley, (B) LIP-Wistar, (C) KA-Sprague-Dawley, and (D) KA-Wistar. (E) Mean stage of spontaneous seizures from different groups, as evaluated from clinical signs according to the Racine scale. Mean seizure grade was obtained from a total of 12 (LIP-SD), 13 (LIP-W), 12 (KA-SD) and 12 (KA-W) spontaneous seizures. (F) Mean duration of video-taped convulsive seizures defined from forelimb automatisms (mean data from 5 seizures per group). (G) Mean duration of electrographic seizures recorded from the dorsal hippocampus. The number of seizures per group is: LIP-SD: 3; LIP-W: 4; KA-SD: 3; KA-W: 4. Data is presented mean ± SD.

Spontaneous seizures were observed in rats treated with systemic injections of both lithium-pilocarpine and kainate weeks before. Clinically, they consisted of brief tonic-clonic seizures with forelimb automatisms with or without rearing and falling. In few animals we confirmed their presence in the dorsal hippocampus using chronic recordings. Electrographically, they were characterized by the appearance of high-amplitude spike-and-wave activity faster than 5 Hz and typically followed by afterdischarges that were associated with the clonic phase of the seizures (Fig. 1A–D). We found similar seizure stages, as evaluated from clinical signs, in animals from the four epileptic groups with no apparent differences in seizure severity (Fig. 1E; Kruskal-Wallis: Chi-2 = 3.005, p = 0.391) and similar duration (Fig. 1F; Kruskal-Wallis: Chi-2 = 0.159, p = 0.984). Similarly, we found no differences in the duration of electrographic seizures (Fig. 1G, Kruskal-Wallis: Chi-2 = 0.524, p = 0.914). Seizure rate was highly variable within and between animals, probably reflecting uneven clustering [20], [21]. However, we observed similar percentage of LIP- and KA-treated animals having daily seizures (KA-W 4/9, KA-SD 2/6, LIP-W 2/6, and LIP-SD 4/9) and having seizures over the course of behavioral tests being therefore suspended for at least two hours (KA-W 5/12, KA-SD 4/16, LIP-W 3/11, and LIP-SD 4/12). Thus both models produced similar epileptic phenotypes in clinical terms, in agreement with previous data [20], [21].

### Different expression of spatial learning deficits in the two experimental models of TLE

We then examined the differences between the two experimental models of TLE on cognitive abilities known to depend on the hippocampal function using the MWM task. In this task, animals learn to locate a submerged platform in a pool by orienting themselves using distal visual cues. Due to previously reported conflicting results [26], [27], [29]–[31], [39], we chose to conduct a cued learning task to train the animals on the procedures required for the MWM task. A repeated-measures ANOVA on escape latencies indicated a significant effect for groups (F(5,77) = 89.463, p<0.0001), for trials (F(6,72) = 77.163, p<0.0001) and for their interaction (F(30,290) = 7.622, p<0.0001), suggesting differences between groups in the ability to find the platform over the course of several trials (data not shown). Control and KA-treated rats from both strains met the learning criterion after few training days, i.e. rats found the flagged platform in less than 20 seconds in two consecutive trials. The control group from both strains reached the criterion on the second training day, while KA-treated Sprague-Dawley and Wistar rats required 3 and 4 days of training, respectively. In contrast, LIP-treated animals from both strains were unable to find the platform after 5 training days. This poses serious concerns about the validity of the MWM task to discriminate for deficits in spatial learning in the LIP model, since –except for the spatial component- cued learning requires similar basic abilities, strategies and escape motivation than the spatial version of the task.

**Figure 2:**
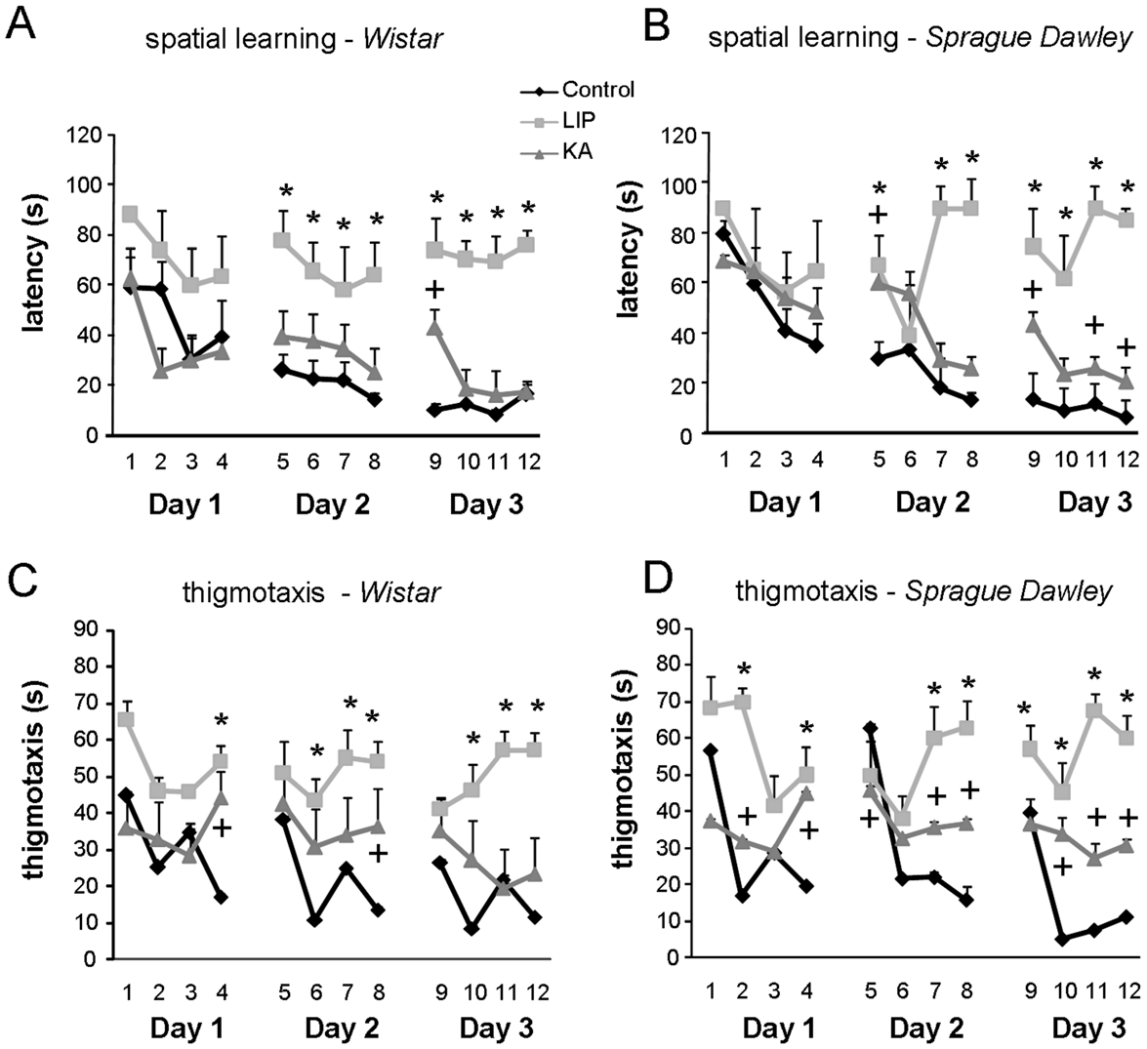
Spatial learning in the Morris water maze. (A) Escape latency for lithium-pilocarpine- (LIP) and kainate- (KA) treated rats of the Wistar strain. (B) Escape latency for LIP- and KA-treated rats compared with controls of the Sprague-Dawley strain. (C) Thigmotactic swimming was evaluated for all groups of the Wistar strain. (D) Thigmotactic swimming in Sprague-Dawley rats. * represents statistical differences at p<0.05 between the LIP and control groups for the corresponding trial. + represents statistical differences at p<0.05 between the KA and control groups for the corresponding trial. See text for detailed p values in each case. The number of animals per group is: Control-W n = 23; Control-SD n = 9; KA-W n = 12; KA-SD n = 16; LIP-W n = 11; LIP-SD n = 12.

Once control and KA-treated rats reached similar levels of procedural learning in the cued task we tested them in the standard MWM acquisition task (Fig. 2). We also included the LIP-treated group to further confirm previous results after failure of cued learning. A repeated-measures ANOVA on escape latencies revealed significant effects for groups (F(5,75) = 52.007, p<0.0001), trials (F(11,65) = 29.351, p<0.0001) and the interaction between groups and trials (F(55,304) = 3.206, p<0.0001). Similar results were obtained when data was re-analyzed by excluding 3 rats that experienced a seizure within an hour after completing the test (pre-seizure effect): significant effects for groups (F(5,72) = 48.944, p<0.0001), trials (F(11,62) = 36.858, p<0.0001) and the interaction between groups and trials (F(55,290) = 3.438, p<0.0001). Post hoc analyses confirmed significant differences in escape latency between the control and LIP groups in trials on day 2 and 3 for both Wistar (Fig. 2A, all at p<0.05) and Sprague-Dawley rats (Fig. 2B, all at p<0.05). This result further confirms the inability of LIP-treated animals to learn the standard MWM tasks probably due to a failure of procedural learning.

Surprisingly, Wistar KA-treated rats did not exhibit differences with the control group except on the first trial of the third day (Fig. 2A, trial 9, p<0.001), suggesting that, in spite of their epileptic condition, they have preserved abilities to form a spatial map by using distal cues. In contrast, Sprague-Dawley KA-treated rats performed poorly in training days 2 (trial 5, p<0.05) and 3 (trials 9, 11 and 12, all at p<0.05, Fig. 2B), indicating that while these animals were able to learn the task they did not perform as good as controls. We also examined the swimming paths over the course of training in the MWM by looking at the thigmotactic behavior (Fig. 2C,D). There was a clear evolution of the thigmotactic index for those groups that successfully learn the task versus the poorest performers. A repeated-measures ANOVA on the thigmotactic behavior revealed significant effects for groups (F(5,69) = 22.353, p<0.0001), trials (F(11,59) = 63,135, p<0.0001) and their interaction (F(55,276) = 12.011, p<0.0001). Post hoc analyses indicated that LIP-treated animals exhibited differences with the control group in several trials for both the Wistar (4, 6, 7, 8, 10, 11 and 12; all at p<0.05) (Fig. 2C) and Sprague-Dawley strains (2, 4, 7,8, 9, 10, 11 and 12, all at p<0.05) (Fig. 2D). Similarly, Sprague-Dawley KA-treated rats presented differences when compared with control in several trials (2, 4, 5, 7, 8, 10, 11 and 12; all at p<0.05) (Fig. 2D) while Wistar KA-treated rats performed similar to control except for trials 4 and 8 (p<0.05). Indeed, we found a positive correlation between the escape latency averaged over the three training days and thigmotactic swimming (r = 0.736, P<0.0001), suggesting that animals that could not solve the task engaged in high thigmotactic behavior. Interestingly, the poor performance of Wistar KA-treated rats on trial 9 (Fig. 2A) cannot be explained by their thigmotactic behavior, which was similar to control for that trial (Fig. 2C), suggesting that a failure for retention might be responsible for the observed deficit.

### Long-term spatial memory is differently affected in LIP- and KA-treated epileptic rats

The data described above suggest a different impairment of spatial learning between KA-treated rats depending on the strain used. Wistar KA-treated rats performed better than their Sprague-Dawley peers in the acquisition phase of the MWM task. However, a poor performance on the first trial of the second and third days suggests that both epileptic animals have difficulties in either retrieving the stored memory and/or forming a strong long-term memory regarding the position of the platform at the beginning of the training procedures. The fact that their performance rapidly improved on subsequent trials over training days 2 and 3, might support a retrieval defect. We thus checked for the strength of long-term spatial memory by using a probe trial, i.e. free swimming without platform, on day 4 after acquisition.

**Figure 3:**
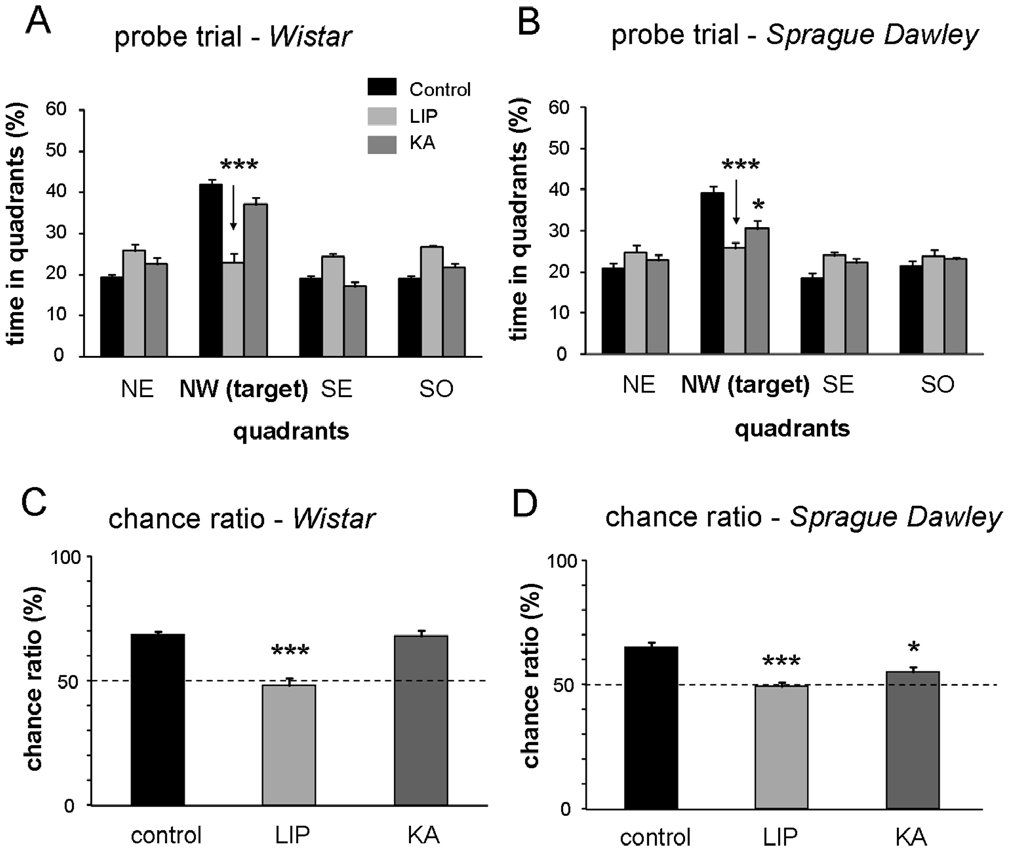
Results of a probe trial designed to measure spatial memory and retrieval capabilities. (A) Time in quadrants for LIP- and KA-treated rats compared with controls of the Wistar strain. The target quadrant is north-west (NW). Other quadrants are: north-east (NE), south-east (SE) and south-west (SW) (B) Time in quadrants for LIP- and KA-treated rats compared with controls of the Sprague-Dawley strain. (C) Analysis of the percent time spent in the target quadrant over the total time spent in the target and the opposite quadrant to test against the chance level of 50% for Wistar rats. Only LIP-treated animals performed at chance level. (D) Analysis of the time spent in the target quadrant against the chance level for Sprague-Dawley rats. Both LIP- and KA-treated rats (*) performed at chance level. The number of animals per group is: Control-W n = 23;Control-SD n = 9; KA-W n = 12; KA-SD n = 16; LIP-W n = 11; LIP-SD n = 12. * represents statistical differences versus control at p<0.05. *** represents statistical differences versus control at p<0.001.

When tested in the probe trial, control animals from both strains spent more time searching for the platform in the target quadrant NW than in others (Fig. 3A,B). A one-way ANOVA confirmed significant differences between groups (F(5,42) = 18.111, p<0.0001). When compared with controls, post hoc analyses indicated significant differences for both LIP-treated (Wistar: p<0.0001; Sprague-Dawley: p<0.0001) and Sprague-Dawley KA-treated rats (p<0.05). In contrast, Wistar KA-treated rats successfully discriminated the NW quadrant and presented no difference with the control group (p = 0.541; Fig. 3A), indicating a focused navigation strategy. Both Wistar and Sprague-Dawley LIP-treated rats and Sprague-Dawley KA-treated animals did not discriminate the target quadrant against a chance level being different than control animals (Fig. 3C,D). While results in LIP-treated animals are not surprising given their failure in the earlier phase of the task, long-term memory impairment of Sprague-Dawley KA-treated rats suggest an increased susceptibility to develop hippocampus-related cognitive deficits in this strain compared to Wistar. What is the nature of these differences between the experimental models of TLE?

### Evaluation of anxiety related-behavior in LIP- and KA-treated epileptic rats

Anxiety is one factor that has been proposed to account for part of the variability in learning and memory [40]. We asked whether possible differences in anxiety related-behavior concur to affect performance in spatial reference learning tasks.

**Figure 4:**
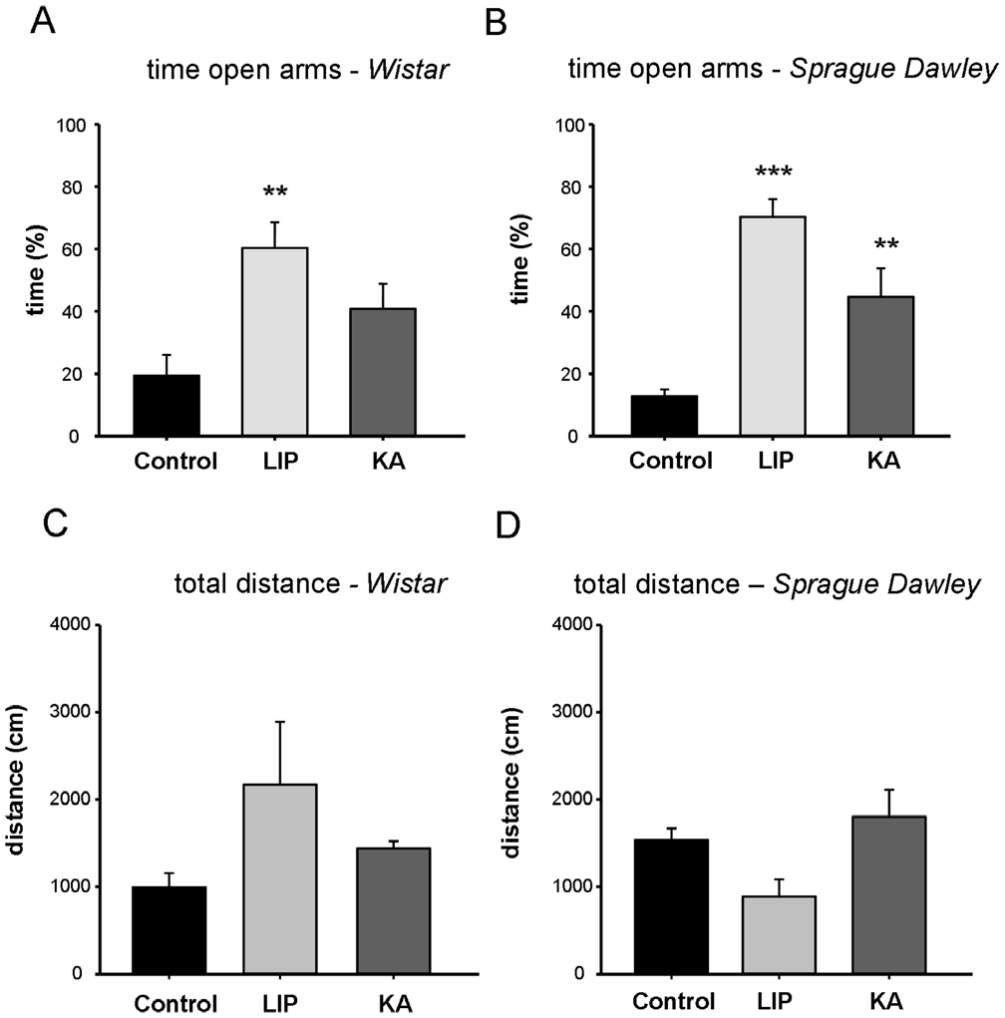
Results obtained on the elevated plus maze. (A) Time spent in the open arms for lithium-pilocarpine- (LIP) and kainate- (KA) treated rats of the Wistar strain. (B) Time spent in the open arms for lithium-pilocarpine- (LIP) and kainate- (KA) treated rats of the Sprague-Dawley strain. (C) Total distance travelled in the maze for the Wistar groups. (D) Total distance travelled in the maze for the Sprague-Dawley groups. Statistical differences are reported at different significance level for comparisons against the control group: ** p<0.01 and *** p<0.001. The number of animals per group is: Control-W n = 23; Control-SD n = 9; KA-W n = 12; KA-SD n = 16; LIP-W n = 10; LIP-SD n = 11.

A one-way ANOVA of the percentage of time spent in the open arms of the EPM indicated significant differences between groups (F(5,54) = 8.923, P<0.001). Post- hoc analyses revealed that the percentage of time spent on the open arms was significantly higher for LIP-treated rats compared with control in both Wistar (p<0.01) and Sprague-Dawley strains (p<0.001) (Fig. 4A,B). A bias for open arm exploration also reached significance in Sprague-Dawley (p<0.01, Fig. 4B) but not in Wistar KA-treated rats (p = 0.372, Fig. 4A) when compared with their respective control. These results indicate decreased anxiety levels in LIP- (Wistar and Sprague-Dawley) and Sprague-Dawley KA-treated rats, and a non-significant trend in Wistar KA-treated animals. Differences between strains in the KA-treated group did not result from differences in activity levels because they travelled similar total distance in the maze (Fig. 4C,D; F(5,54) = 1.577, P = 0.184). Altogether, the behavioural tests reveal major differences between LIP- and KA-treated epileptic rats regarding spatial memory deficits and anxiety.

### Different lesional patterns in LIP- and KA-treated epileptic rats: MRI data

Previous reports reveal a larger extension of brain damage detected 24 hours after systemic administration of pilocarpine compared with kainate [25]. We therefore reasoned that such difference would persist in the chronic phase rendering LIP-treated rats more severely disrupted in cognitive tasks than KA-treated animals. We also wondered whether a different magnitude of brain damage in Wistar versus Sprague-Dawley rats after kainate injection might be related to the behavioral differences observed in our study. We thus performed volumetric MRI studies, histology and neuronal immunostaining to reveal differences between the two experimental models of TLE.

**Figure 5:**
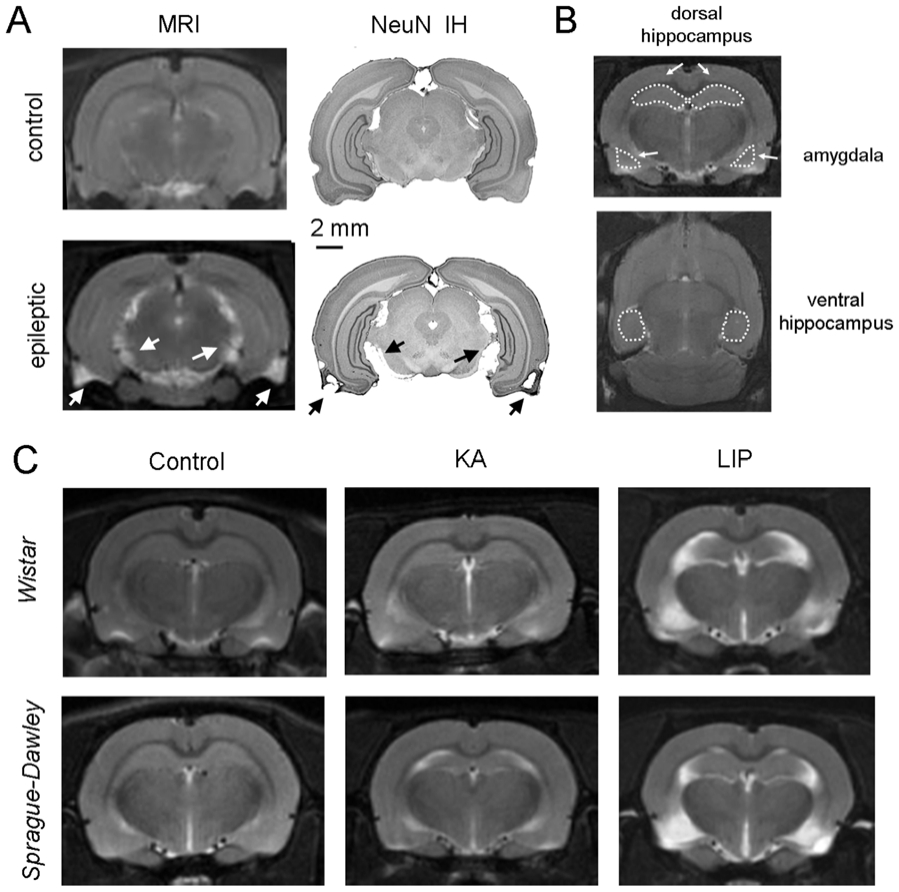
Magnetic resonant imaging (MRI). (A) T2-weighted MRI was used to look at structural differences between experimental groups. We identified different brain structures by directly comparing with the rat brain atlas and with equivalent sections stained against the neuronal protein NeuN (NeuN-IH). Note the higher signal intensity in MRI from epileptic animals corresponding to lesions as identified in the NeuN sections (arrows). (B) Coronal and horizontal MRI sections were used to outline regions of interest (ROIs) at different structures such as the dorsal and the ventral hippocampus and the amygdala. (C) Representative MRI images of coronal sections from the different experimental groups. High MRI signal intensity is evident in LIP-treated rats from both strains. See Table 1 for quantification.

T2-weighted MRI was used to elucidate structural changes present in the brain of epileptic rats. MRI scans provided good contrast to identify different brain structures when directly compared with the rat brain atlas and with equivalent sections stained against the neuronal protein NeuN (Fig. 5A). In epileptic animals, lesions can be directly identified by the higher MRI signal intensity reflecting water increase (Fig. 5A, arrows). We used coronal sections to manually outline regions of interest (ROIs), including part of the dorsal hippocampus and the lateral and basolateral amygdala from both hemispheres (Fig. 5B; see Methods). The partial volume of perirhinal and piriform cortices was also calculated from these sections. Horizontal sections were used to measure ROI volumes in part of the ventral hippocampus and the entorhinal cortex (Fig. 5B).

MRI data clearly showed larger lesions in LIP- than in KA-treated animals for both strains, mostly affecting the hippocampus, the lateral and basolateral amygdala and the more ventral aspect of the neocortex (Fig. 5C). Volumetric analysis revealed significant changes in the relative sizes for most ROIs analyzed in control versus LIP-treated rats (Table 1), in good agreement with previous data obtained 24 hours after the *status* [25]. Interestingly, no significant differences were found in the relative volume of the hippocampus of Wistar KA-treated rats when compared to controls. Instead, Sprague-Dawley KA-treated animals showed significant decrease of the dorsal and ventral hippocampus when compared to controls (Table 1), suggesting larger hippocampal atrophy than in Wistar animals. Indeed, ANOVA revealed an effect for groups (F(5,33) = 32.996, p<0.001 for the dorsal hippocampus, F(5,33) = 37.296, p<0.001 for the ventral hippocampus), confirming a different extension of multifocal brain damage.

**Table 1:** Volumetric analysis of MRI region of interest (ROI) data (mean ± SD).

Region of interest (ROI)	Relative volumen (%)			Change (%)	
	Control W	KA W	LIP W	KA vs. Control	LIP vs. control
Basolateral Amygdala	2,39±0,12	2,27±0,17	**0,00** ***	-5,0	-100
Dorsal Hippocampus	7,78±0,24	6,87±0,76	**3,53±0,44** ***	-11,7	-54,6
Ventral Hippocampus	4,18±0,12	3,76±0,60	**1,81±0,19** ***	-10,0	-57,1
Perirhinal Cortex	2,93±0,08	2,40±0,30	**0,00** ***	-18,0	-100
Piriform Cortex	2,18±0,18	1,98±0,62	**0,00** ***	-9,0	-100
Entorhinal Cortex	3,33±0,41	3,11±0,26	**1,75±0,12** ***	-6,6	-47,4
n	6	7	6		

	Control SD	KA SD	LIP SD		
Basolateral Amygdala	2,63±0,37	1,96±0,73	**0,00** **	-25,0	-100,0
Dorsal Hippocampus	8,52±0,54	**4,94±1,04** ***	**3,47±1,35** ***	-42,0	-58,1
Ventral Hippocampus	4,28±0,19	**3,04±0,34** ***	**2,37±0,42** ***	-28,9	-42,9
Perirhinal Cortex	2,45±0,28	2,48±0,47	**0,00** ***	+1,2	-100,0
Piriform Cortex	2,14±0,19	1,62±0,28	**0,00** ***	-24,0	-100,0
Entorhinal Cortex	2,88±0,38	3,11±0,29	2,93±0,42	+8,0	-1,7
n	5	10	8		

W: Wistar, SD: Sprague-Dawley, KA- kainic acid, LIP: lithium-pilocarpine. Statistical differences are reported at different significance level for comparisons against the control group (Tukey's HSD test):

**p<0.01 and

***p<0.001. Statistical differences are highlighted.

### Different lesional patterns in LIP- and KA-treated epileptic rats: stereological cell counting

**Figure 6:**
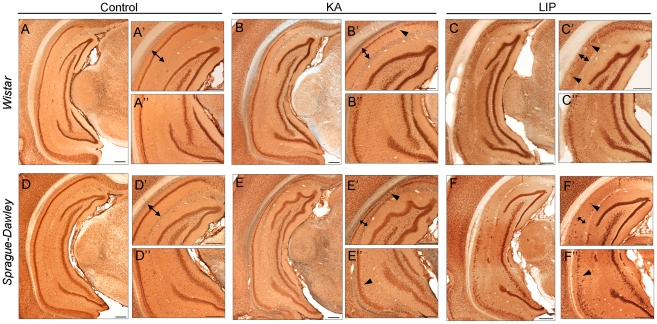
NeuN immunostaining was used to qualitatively validate differences between different experimental groups. (A) Coronal sections corresponding to about -5.4 mm from bregma showing the dorso (top) to ventral (bottom) extension of the hippocampus from a normal Wistar rat. (A') Details of the dorsal hippocampus shown in A. The double-headed arrow marks the extension of the CA1 region from the *alveus* to the hippocampal fissure. (A″) Details of the ventral aspect of the hippocampus shown in A. (B) Similar coronal section from a representative KA-treated rat from the Wistar strain. (B') Details of the dorsal hippocampus. Note the reduction of the CA1 region (double-headed arrow) when compared with control rat in A'. Note also cell loss in the CA1 region (arrowhead). (B″) Details of the ventral hippocampus shown in B. (C) Coronal section from a representative LIP-treated rat from the Wistar strain. Note the larger atrophy when compared with the control and KA-treated rats. (C') Details of the dorsal hippocampus. Note the reduction of the CA1 region (double-headed arrow) and massive cell loss in the CA1 region and the hilus (arrowhead). (C″) Details of the ventral hippocampus shown in C. (D,E,F) Same as above for the Sprague-Dawley strain. All scale bars are 500 µm.

We then used immunostaining and histological techniques to further validate differences between TLE models and strains. NeuN immunostaining confirmed large neuronal loss in both the dorsal and the ventral hippocampus of LIP-treated rats of both strains (Fig. 6A,D versus Fig. 6C,F), mostly affecting the CA1 region (Fig. 6C,C″,F',F″'). KA-treated rats from both strains appeared less affected than LIP-treated animals (Fig. 6B,E versus Fig. 6C,F), but CA1 neuronal loss was apparent when compared with control rats (Fig. 6B',B″,E',E″). Interestingly, larger hippocampal atrophy of the CA1 region was evident in Sprague-Dawley KA-treated rats compared with Wistar (Fig. 6B' versus Fig. 6E'), further confirming MRI data. Unbiased stereological cell counting confirmed significant cell loss in the CA1 region of both KA- and LIP-treated animals from both strains, with variability between animals (Table 2). In the CA3 area, cell loss in both epileptic groups was significant for Wistar but not for Sprague-Dawley rats, suggesting a strain difference as reported previously in mice [41] and rats [42].

**Table 2:** Unbiased stereological estimates of total neurons in the dorsal hippocampus and the basolateral amygdala (mean ± SD).

	Wistar				Sprague-Dawley			
Region/group	Total (×10^3^)	Volume (mm^3^)	Density (×10^3^/mm^3^)	n	Total	Volume (mm^3^)	Density (×10^3^/mm^3^)	n
CA1: control	35.27±2.59	0.145±0.014	244.28±7.53	3	39.61±5.06	0.149±0.010	267.14±43.03	3
CA1: kainate	**11.30±4.31** *	**0.070±0.027** *	**161.47±2.87** *	4	**20.82±12.33** *	0.136±0.017	148.21±70.88 *	3
CA1: lithium-pilocarpine	**17.87±6.94** *	0.106±0.040	189.85±91.72	4	**19.19±10.03** *	0.133±0.012	147.07±87.44	3
CA3: control	41.01±8.28	0.408±0.072	100.34±3.32	3	33.57±5.72	0.379±0.033	88.05±9.12	3
CA3: kainate	**24.42±4.27** *	**0.250±0.050** *	98.05±4.19	3	32.41±3.55	0.374±0.034	86.30±3.08	3
CA3: lithium-pilocarpine	**26.04±5.55** *	0.397±0.063	**65.27±8.55** *	3	38.40±2.96	0.436±0.042	88.84±14.36	3
Amygdala: control	154.64±23.84	2.366±0.142	65.49±10.90	3	154.12±24.86	2.141±0.134	72.10±11.51	3
Amygdala: kainate	141.26±60.03	1.848±0.607	75.11±18.33	3	**100.07±17.63** *	1.738±0.125 *	58.17±14.04	3

Statistical differences are reported at different significance level for comparisons against the control group (Mann-Whitney test):

*p<0.05. Statistical differences are highlighted.

**Figure 7:**
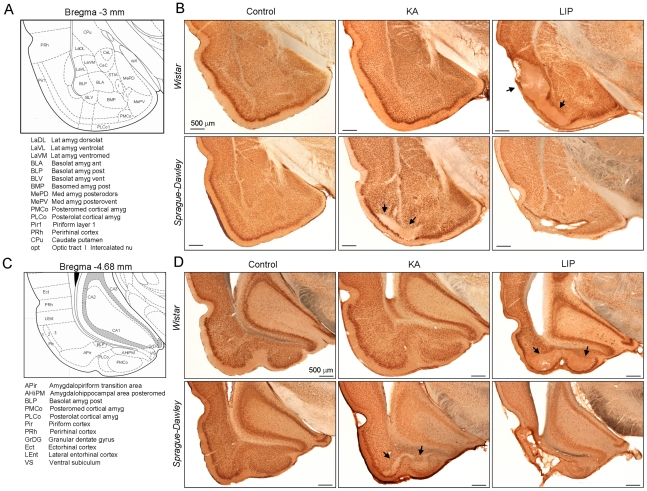
Detailed analysis of the histoarchitecture of several amygdaloid nuclei. (A) Coronal sections at about -3 mm from bregma were used to look at the basal nuclei of the amygdala. Different nuclei are identified using the stereoataxic atlas (Paxinos and Watson 2005). (B) NeuN immunostained sections from control, KA- and LIP-treated rats in both strains, Wistar and Sprague-Dawley. Note the large shrinkage of the basolateral amigdala of LIP-treated rats and lesions in Sprague-Dawley but not in Wistar KA-treated rats. (C) Coronal sections at about -4.7 mm from bregma were used to look at the posterocortical nuclei of the amygdala. Different nuclei are identified using the stereoataxic atlas (Paxinos and Watson 2005) (D) NeuN immunostained sections from control, KA- and LIP-treated rats in both strains, Wistar and Sprague-Dawley. All scale bars are 500 µm. Arrows indicate lesional regions at different nuclei.

We also examined the histoarchitecture of the amygdala by using coronal sections corresponding to about -3 mm (Fig. 7A) and -4.7 mm (Fig. 7C) from bregma to focus on the basolateral and posterocortical amygdaloid nuclei, respectively. LIP-treated animals of both strains were severely affected with large lesions in the different nuclei (Fig. 7B,D). We found obvious differences between strains with Sprague-Dawley KA-treated rats exhibiting larger neuronal loss in both the basal and the posterocortical nuclei (Fig. 7B,D) than controls and the Wistar KA group. Unbiased stereological cell counting of the lateral and basolateral amygdala confirmed significant cell loss in KA-treated Sprague-Dawley rats, with Wistar KA animals being not different from controls (Table 2). This drop of the number of cells was poorly reflected in density values due to the compensatory effect of a reduced volume (Table 2). At the caudal level, neuronal loss was visible in the amygdalo-hippocampal area which is densely connected with the basomedial amygdala and the ventral hippocampus (Fig. 7D, arrows). These strain differences in KA-treated rats, which were not apparent in MRI probably due to its poor resolution to discriminate between amygdaloid nuclei, might also account for performance alterations of Sprague-Dawley KA-treated rats in the EPM.

### Different lesional patterns in LIP- and KA-treated epileptic rats: mossy fiber sprouting

**Figure 8:**
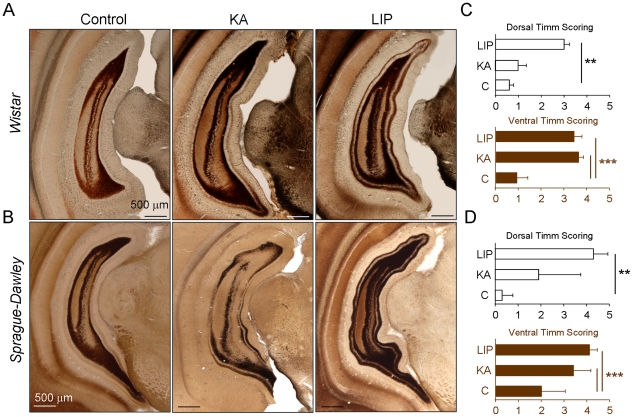
Timm staining showing mossy fiber sprouting (MFS) present in epileptic animals. MFS is one of the neuropathological hallmarks of human temporal lobe epilepsy and consist in the sprouting of mossy fiber reinnervating the dendrites of granular cells. A, Representative Timm stained sections for Wistar rats. B, Representative Timm stained sections in Sprague-Dawley rats. MFS was present both at the ventral (bottom) and the dorsal (top) dentate gyrus of the hippocampus of LIP-treated animals, while it was only observed ventrally in KA-treated rats. All scale bars are 500 µm. C, Quantification of MFS at the dorsal and the ventral hippocampus for the Wistar groups (data from n = 3 control, n = 5 KA and n = 2 LIP treated animals). Timm staining was quantified in a scale from 0 (no MFS) to 5 (confluent dense laminar band of Timm granules extending into the inner molecular layer). D, MFS Timm scores at the dorsal and the ventral hippocampus from the Sprague-Dawley groups. Data from n = 2 control, n = 3 KA- and n = 3 LIP-treated rats. Statistical differences are reported at different significance level for comparisons against the control group: * p<0.05, ** p<0.01 and *** p<0.001.

Finally, we noted differences in the pattern of mossy fiber sprouting (MFS) between LIP- and KA-treated animals from both strains as assessed by Timm staining (Fig. 8A,B). The extent of mossy fiber sprouting was evaluated by rating the distribution of supragranular Timm granules in the dorsal and the ventral hippocampus in a scale from 0 to 5 [38]. MFS was present both at the ventral and the dorsal dentate gyrus of the hippocampus of LIP-treated animals, while it was only observed ventrally in KA-treated rats (Fig. 8C,D). Even though the role of MFS in seizures is controversial [43], this difference suggests a more normal intrinsic connectivity at the dorsal dentate gyrus of KA- than LIP-treated rats.

### Relationship between indices of spatial memory deficits, anxiety and brain lesions

Altogether our data confirm differences between the lithium-pilocarpine and kainate models of TLE and further uncover strain differences between Wistar and Sprague-Dawley rats when treated with kainic acid. Given the cognitive differences between groups in the acquisition and retention phases of the MWM tasks, we wondered whether there is a correlation between different anxiety levels and the behavioral and lesional indices that could explain spatial memory deficits.

**Figure 9:**
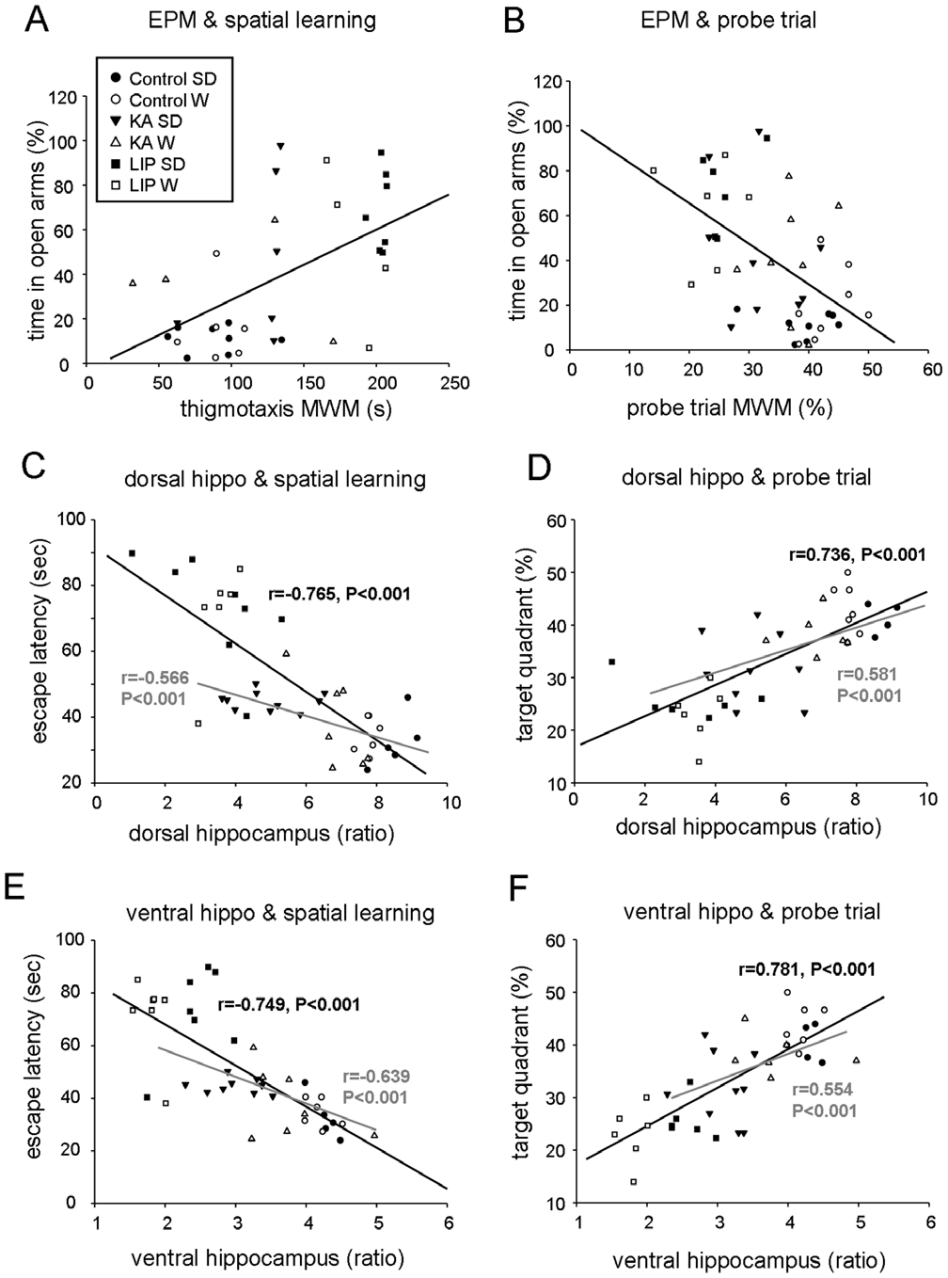
Statistical correlation between indices of spatial memory deficits, anxiety and brain lesions. (A) Correlation between the thigmotactic swimming in the water maze and the time spent in the open arms of the elevated plus maze (EPM) for all the experimental groups. See legend for symbol code. (B) Correlation between the results on the probe trial and the time spent in the open arms of the EPM. See legend in A for the symbol code. (C) Correlation between the relative volumen of the dorsal hippocampus and the escape latency to the hidden platform in the water maze tasks. (D) Correlation between the relative volumen of the dorsal hippocampus and the results on the probe trial. (E) Correlation between the relative volume of the ventral hippocampus and the escape latency to in the water maze tasks. (F) Correlation between the relative volumen of the ventral hippocampus and the results on the probe trial. In all cases, black line and values represent the correlation for all groups included. Gray line and values represent correlation obtained when the LIP group was excluded.

We found a positive correlation between the time spent in the open arms of the EPM and the thigmotactic behavior in the pool (r = 0.552, P<0.001; Fig. 9A), indicating that the strategies typically used to find the submerged platform might be hampered by changes in anxiety. Moreover, a negative correlation between the time in the open arms and the time spent in the target quadrant of the pool during the probe trial (r = −0.555, P<0.01; Fig. 9B), might further suggest a modulatory role of anxiety-related behaviors. In both cases, the observed correlations indicated that the lower the anxiety (i.e., the higher the time spent in the open arms of the EPM), the higher the behavioral deficits in the water maze, as illustrated by higher thigmotactic swimming and lower time spent in the target quadrant (e.g., both parameters indicating a lack of focused platform searching). However, this is opposed to the expected relationship according to the literature in normal animals, with higher anxiety being classically linked to impaired water maze performance [40], [44]. In fact, this effect did not persist when the LIP- group was excluded (r = 0.263, P = 0.225 for EPM and thigmotactic behavior and r = −0.360, P = 0.047 for EPM and the probe trial). Similarly, the time spent in the open arms and the volume of part of the amygdala as estimated from the ROI outlined at coronal sections of MRI scans were also negatively correlated when all groups were included (r = −0.582, P<0.01), but not when the LIP group was left out (r = −0.258, P = 0.185; data not shown), pointing to a large bias introduced by the overall poor performance of these animals. Indeed, there was high correlation between ROIs outlined in the dorsal and the ventral hippocampus and the amygdala (r = 0.804, P<0.00001, amygdala vs dorsal hippcampus; r = 0.833, P<0.00001 amygdala vs ventral hippocampus), further suggesting that the behavioral interactions discussed above might be rather reflecting an underlying correlation of the lesional pattern.

We also wondered about the possible relationship between indices in the MWM task and brain lesions by using MRI data. We found a strong negative correlation between the escape latency averaged during the three training days of the MWM and the relative volume of the dorsal (r = −0.765, P<0.0001; Fig. 9C) and the ventral hippocampus (r = −0.749, P<0.0001; Fig. 9E). Similarly, a correlation was present between performance in the probe trial of the MWM task and the volume of the dorsal (r = 0.710, P<0.0001; Fig. 9D) and the ventral hippocampal ROIs (r = 0.772, P<0.000; Fig. 9F). These relationships were also observed when the LIP group was excluded (Fig. 9C–F), suggesting that the spatial memory deficits could be explained by the reduced hippocampal volume.

## Discussion

One major conclusion of our study is that there are differences between LIP- and KA-treated epileptic rats regarding spatial memory deficits, anxiety and multifocal damage affecting different brain structures. We found that Wistar KA-treated rats, which developed spontaneous seizures and presented the major neuropathological features of TLE (hippocampal cell loss and MFS), successfully learned and remembered a hippocampal-dependent task in the Morris water maze. In contrast, Sprague-Dawley KA-treated rats performed poorly at the acquisition phase of the task and failed for retention, which concurred with lower anxiety levels in the elevated maze and larger brain damage compared with Wistar animals. LIP-treated rats dramatically failed in the MWM and spent more time in the open arms of the EPM, which concurred with severe multifocal brain lesions. Hence, our results strongly indicate that the formation and retention of spatial memories, as tested in the water maze, is not necessarily affected in TLE and that comorbidity between spatial deficits and anxiety seems to be more related with lesions affecting the amygdala than with the epileptic condition *per se*.

One possibility to explain disparities between models and strains is related with differences in the severity of epilepsy. It is well known that seizures and interictal activity directly affect cognitive performance [4]–[9], [45] and that frequent seizures can produce larger damage [46]. However, we found no clear differences regarding seizure grade and duration between groups. Recent reports using long-term continuous video-EEG monitoring of both pilocarpine and KA-treated rats have proved that spontaneous seizures occur in clusters with large variability between animals [20], [21]. While possible differences on the seizure clustering cycle between groups might account for the behavioural differences reported here, the existence of similar percentage of animals having daily seizures in each group further suggest they were comparable in terms of seizure severity. Indeed, the distinct lesional pattern in LIP- versus KA-treated animals already occurs 24 h after the status [25], further suggesting that behavioural differences truly reflect strain and model features [42], [47].

The emotional state is known to physiologically modulate learning and memory [40], [44], [48], [49]. In TLE, stress and anxiety disorders coexist with the epileptic condition to exacerbate cognitive deficits [14]–[17]. However, there are open questions regarding the neurobiology of this comorbidity. We found reduced anxiety levels in epileptic rats with clear damage of the amygdala, as observed both at the basolateral and posterocortical nuclei, but not in animals with intact histoarchitecture of these nuclei. Indeed, the lack of alterations in the amygdala of Wistar epileptic KA-treated rats and their normal performance in the EPM further suggests a functional separation between the TLE condition and anxiety-related disorders. This is in agreement with previous data showing that full kindling of the dorsal hippocampus does not affect anxiety-related behaviour [50], whereas amygdaloid kindling can be anxiogenic or anxiolitic depending on the baseline anxiety levels [51], laterality [52] and the kindled site [53]. According to this data, there is no logic behind the bilateral indiscriminate damage of the basal and posterocortical amygdaloid nuclei and the reduced anxiety detected in the epileptic groups. Indeed, the negative correlation we found between the anxiety-like behaviour in the EPM and thigmotactic behaviour in the water maze is opposed to the expected relationship in normal animals [40], [44], suggesting that the formerly described relationship does not account when low anxiety levels are coupled to a (psycho)pathological process. A possible explanation for the altered anxiety-related behaviour can be that amygdalar lesions and ventral hippocampal dysfunction both affect the corticosterone response to stress in the tasks [54], [55]. This is supported by our unpublished data pointing to a post-training increase of plasmatic corticosterone in LIP-treated but not in KA-treated animals, in agreement with previous reports for the pilocarpine model [56].

Previous reports of spatial memory deficits in experimental TLE were performed using the pilocarpine model [26]–[29], known to produce substantial multifocal damage 24 hours after the *status epilepticus* [25]. In contrast, kainate injections induce less acute damage with brain areas being affected at lower intensity than in pilocarpine-treated rats [25]. Our data further confirm that such a model-difference persists for weeks after the *status* and it seems to run in parallel with the animal's ability to perform the tasks required to evaluate spatial cognitive deficits. Indeed, LIP-treated animals were unable to learn a cued version of the water maze posing doubts on the validity of this task to discriminate for specific deficits of spatial learning in this model. Such impairment could be related with visible shrinkage of the caudate nucleus in MRI data from LIP- but not in KA-treated animals (not shown). In addition, evidence supports a role for the hippocampus in the procedural learning aspects of water maze navigation [57], which might also explain cued-learning failure of these rats.

We also found strain differences between Wistar and Sprague-Dawley rats after kainate-induced *status*. The genetic background is known to affect the expression of epileptic phenotypes and to underlie different sensitivity for seizures in mice and rats [42], [58], [59]. Systemic injections of kainic acid produce hippocampal cell death in some but not all mouse [41] and rat strains [42]. Also, an effect of strain differences in learning and memory capabilities have been reported not only for normal animals [60], [61], but also for the pilocarpine model in Long Evans and Wistar rats [39] and in mice [58]. We propose that model and strain differences can be further exploited to validate TLE models better suitable for cognitive studies in epilepsy.

The most distinctive type of cognitive impairment in human TLE with hippocampal sclerosis is related to a failure in the long-term retrieval of newly acquired information [11], [62]. This comprises a deficit of episodic memory, which is postulated to be hippocampal-dependent [63]–[65], but not of semantic neither spatial memory as such [11], [66]. Wistar KA-treated rats showed learning capabilities similar to control in the three days of acquisition of the MWM task. Only at the first trial of the third training day performance was poorest, suggesting they might have difficulties to retain and/or to get access to formerly learnt information for the position of the hidden platform. It is therefore possible that mild long-term memory deficits could emerge clearer in these animals when the spatiotemporal contingencies of the task oppose a larger demand of hippocampal function.

The preservation of the ability to form hippocampal-dependent spatial memories in Wistar KA-treated rats strongly suggests that epileptic animals with hippocampal sclerosis in an apparently normal brain do not necessarily experience spatial memory problems. Instead, a mild poor performance in finding the platform on inter-day trials points to deficits of long-term memory as a potential basis for disruption of the episodic memory [67]. This revives the intriguing question that the role of the hippocampus might be more related to an organizational representation of events occurring at different spatiotemporal and contextual sequences than with spatial memory exclusively [68]. Hence, Wistar KA-treated epileptic rats with hippocampal neuronal loss and circuit reorganization might help to better target the type and mechanisms of TLE-associated memory impairment. This is not in contradiction with data showing that seizures and interictal activity negatively impact on spatial memory performance [4], [5], [7], [9]. Indeed, several factors might be interfering with the inter-day ability of these rats to successfully recall the position of the platform, including unsupervised seizures and distortion of the elementary process underlying memory consolidation. It is known that the hippocampal place fields are reconfigured hours after a seizure [45] and that subclinical pathological activity at the hippocampus can produce transient cognitive impairment [6]–[8]. In addition, normal hippocampal rhythms like theta, gamma and ripples, which are proposed to play crucial roles in memory formation and consolidation, might be also affected [28], [69]. Further work is thus required to characterize the nature of cognitive impairment in TLE and to overcome the basic underlying mechanisms. Our data bring the attention to the importance of carefully choosing the appropriate experimental model for this endeavor.
